# Randomized Control Trials Longitudinal assessments of child growth: A six-year follow-up of a cluster-randomized maternal education trial

**DOI:** 10.1016/j.clnu.2021.08.007

**Published:** 2021-08-20

**Authors:** Prudence Atukunda, Moses Ngari, Xi Chen, Ane C. Westerberg, Per O. Iversen, Grace Muhoozi

**Affiliations:** a Department of Nutrition, University of Oslo, Norway; b The Childhood Acute Illness & Nutrition Network (CHAIN), Nairobi, Kenya; c KEMRI/Wellcome Trust Research Programme, Kilifi, Kenya; d Department of Nutrition and Food Hygiene, School of Public Health, Tongji Medical College, Huazhong University of Science and Technology, Wuhan, People’s Republic of China; e Institute of Health Sciences, Kristiania University College, Oslo, Norway; f Division of Obstetrics and Gynecology, Oslo University Hospital, Oslo, Norway; g Department of Haematology, Oslo University Hospital, Oslo, Norway; h Division of Human Nutrition, Stellenbosch University, Tygerberg, South Africa; i Department of Human Nutrition and Home Economics, Kyambogo University, Kampala, Uganda

**Keywords:** Body composition, Growth impairment, Growth velocity, Maternal education, Sub-Saharan Africa, Stunting

## Abstract

**Background & aims:**

Child growth impairments are rampant in sub-Saharan Africa. To combat this important health problem, long-term follow-up studies are needed to examine possible benefits and sustainability of various interventions designed to correct inadequate child growth. Our aim was to perform a follow-up study of children aged 60−72 months whose mothers participated in a two-armed cluster-randomized education intervention trial lasting 6 months in rural Uganda when their children were 6−8 months old with data collection at 20−24 and at 36 months. The education focused on nutrition, hygiene, and child stimulation.

**Methods:**

We measured growth using anthropometry converted to z-scores according to WHO guidelines. We also included assessments of body composition using bioimpedance. We used multilevel mixed effect linear regression models with maximum likelihood method, unstructured variance-covariance structure, and the cluster as a random effect component to compare data from the intervention (receiving the education and routine health care) with the control group (receiving only routine health care).

**Results:**

Of the 511 children included in the original trial, data from 166/263 (63%) and 141/248 (57%) of the children in the intervention and control group, respectively, were available for the current follow-up study. We found no significant differences in any anthropometrical z-score between the two study groups at child age of 60−72 months, except that children in the intervention group had lower (P = 0.006) weight-for-height z-score than the controls. There were no significant differences in the trajectories of z-scores or height growth velocity (cm/year) from baseline (start of original trial) to child age of 60−72 months. Neither did we detect any significant difference between the intervention and control group regarding body composition (fat mass, fat free mass, and total body water) at child age 60−72 months. Separate gender analyses had no significant impact on any of the growth or body composition findings.

**Conclusion:**

In this long-term study of children participating in a randomized maternal education trial, we found no significant impact of the intervention on anthropometrical z-scores, height growth velocity or body composition.

**Trial registration:**

Clinical Trials (clinical trials.gov) ClinicalTrials.gov ID NCT 02098031.

## 1 Introduction

Impaired linear growth in children, i.e. reduced length/height, has for decades been viewed as a proxy for undernutrition. This anthropometric deficiency is termed stunting and is defined as height-for-age z-score (HAZ) more than two standard deviations below the median of the WHO child growth standard [1]. A recent report by the UN Food and Agriculture Organization found that among children below five years worldwide, 144 million (21.3%) were stunted [2]. Although globally there is progress in reducing the number of stunted children [3], the world is behind course to meet the World Health Assembly goal of a 40% reduction to less than 5% by 2025 [4]. Notably, while most interventions to curb stunting has focused on children below 5 years, far less resources have been directed at correcting linear growth deficiencies among older children. Moreover, due to the worrisome food insecurity in sub-Saharan low- and middle-income countries, stunting continues to be rampant in many regions on the African continent [2,5].

The widespread use of stunting as an indicator of linear growth failure and as a predictor of negative health outcomes and mortality later in life, largely stems from its robustness and low cost for use in demanding resource-strained settings. However, stunting alone does not necessarily capture how adverse nutritional exposures affect more refined aspects of growth, impact on the dynamics in child growth over time and on subsequent risk of disease in adolescent and adult life [6]. In addition, stunting does not take into account body composition, a measure that has the potential to better unlock how events in utero, postnatally and early childhood, may shape the nutritional and metabolic health of the individual child [6]. Importantly, body composition, in particular fat vs. lean body mass distribution, may vary among children with similar anthropometrical measures [6]. In addition, ethnic variations in normal child growth patterns might be missed using the WHO growth standard for stunting that is based on growth data from different populations [7]. Also relevant in this context is the recent claim by Scheffler et al. that instead of using stunting as a marker of undernutrition, one should use catch-up growth as indicator of past undernourishment [8].

Primarily to prevent impaired linear growth, we conducted the “Child Nutrition and Development Study” (CHNUDEV) in 2013−14, a two-armed, pragmatic cluster-randomized controlled trial (RCT). In that trial we examined child anthropometrical effects of a maternal education intervention focusing on nutrition, hygiene and child stimulation in South-Western Uganda, a part of the country with high prevalence of stunting [9,10]. The trial included 511 mother−child pairs and started when the children were 6−8 months [11]. We found no significant effect of this intervention on height at child age of 20−24 months [11], but in the intervention group there was a significant reduction in growth faltering when the children were 36 months [12].

Monitoring child growth patterns over time is essential to evaluate long-term effects of interventions given at early child ages. Thus, we have now performed a follow-up of our RCT cohort when the children reached 60−72 months, i.e. at the time of school-start. This unique longitudinal data set allowed description of both anthropometry and growth velocity trajectories. In addition, we collected body composition data at 60−72 months age.

## 2 Methods

### 2.1 Approvals

The RCT was approved by the Uganda National Council for Science (HS 1809), the AIDS Support Organisation Research Ethics Committee (No. TASOREC/06/15-UG-REC-009), and the Norwegian Regional Committee for Medical and Health Research Ethics (no. 2013/1833).

### 2.2 Study setting and participants

The RCT was conducted between October 23, 2013 and February 16, 2014 in the neighbouring districts of Kabale and Kisoro in South-Western Uganda because of high stunting rates [13]. Sample size calculation, enrolment and randomisation of the 511 study participants in the original RCT has been detailed in the Supplementary Methods and elsewhere [11]. Briefly, simple random sampling was performed to allocate 10 sub-counties (clusters) in each district (6 from Kabale and 4 from Kisoro districts) to either the intervention or control group. All villages in each sub-county (intervention or control) were listed alphabetically and computer-generated random numbers were then used to obtain the villages, and finally complete enumeration was used to obtain participating households. Intervention villages did not share common geographical boundaries with control villages to prevent “contamination” of intervention-contents between the two study groups. Exclusion criteria were congenital malformations or physical handicap among children that would influence food intake, growth, mental or brain illness as evidenced by mother or health worker.

### 2.3 Intervention contents

An education intervention emphasizing nutrition, hygiene (including oral hygiene) and stimulation was delivered to mothers in the intervention group as described in the Supplementary Methods and as previously detailed [11]. In short, cooking and oral hygiene demonstrations together with making of play toys to promote child stimulation, were parts of the education intervention package. The intervention lasted six months in which each group of mothers received three main education sessions (with a nutrition education team) followed by monthly village meetings. Thereafter, booster sessions were provided every third month until the age of 36 months (Supplementary Methods). The intervention group received routine health care and the education intervention while the control group received only routine health care. Our strategy with the intervention was to promote behaviour change through providing information and prompt practice (demonstrations).

### 2.4 Anthropometrical measurements

Height, weight, and mid-upper arm circumference at each follow-up sampling time were measured according to WHO guidelines by trained nutritionists as detailed previously [1,11]. Weight (to the nearest 0.1 kg) was measured with a Seca-scale model 881 (Hamburg, Germany), whereas recumbent length was measured (to the nearest 0.1 cm) with a length board (Seca, SO114530). MUAC was measured with a non-stretchable tape (Seca, S0145620 MUAC, Child 11.5 Red/PAC-50) at the midpoint between the acromion and the olecranon. Interobserver Pearson’s correlation coefficients for reliability ranged between 0.91 and 0.98 for all anthropometric measurements. To avoid bias, the team that assessed growth in the RCT and previous follow-up studies was replaced by a new team, which was blinded to group allocation.

Height growth velocity was calculated as the difference between the height values (cm) at each follow-up age (12−16,20−24,36 and 60−72 months) and the baseline height values divided by the follow-up time in years.

### 2.5 Assessment of body composition

Body composition at 60−72 months age was estimated using a dual frequency (6.25 and 50 kHz) bioimpedance analyzer (Tanita DC 430 MA, Hong Kong). The measurements were conducted in the morning among non-fasting children with light clothing.

### 2.6 Statistical methods

Whereas the RCT recruited in total 511 mother/child pairs, the minimum calculated number of such pairs was 352 to achieve the primary outcome, i.e. to detect a difference of 0.3 SD (power 0.80, alpha 0.05, intra-cluster correlation coefficient 0.01) in HAZ at 20−24 months of age between the intervention and control group as described in the Supplementary Methods and elsewhere [11]. The current study used data from the available 307 children that could be assessed at 60−72 months of age.

The anthropometric z-scores at baseline (when the RCT started with the children aged 6−8 months) and when the children reached 60−72 months of age, were calculated using the 2006 and 2007 WHO growth references, respectively. We computed the following z-scores as ([observed value − median value of the reference population]/SD value of the reference population) for height-for-age (HAZ), weight-for-age (WAZ), weight-for-height (WHZ) and mid-upper arm circumference-for-age (MUACZ). Since the 2007 WHO growth reference does not have MUACZ for age references, we used the method of Mramba et al. to calculate MUACZ at 60−72 months of age [14]. Underweight was defined as WAZ < −2 whereas wasting was defined as WHZ < −2 for children 6−8 months (baseline) and as BMI-for-age z-score < −2 for children aged 60−72 months (current follow-up study).

The analyses used the intention-to-treat approach and all the statistical tests were two-sided. We calculated anthropometric and body composition means (95% confidence intervals) for each study group (intervention and control) at 60−72 months of age and their cluster-adjusted mean differences. We used multilevel mixed effect linear regression models with maximum likelihood method, unstructured variance-covariance structure and the cluster as a random effect component to compare the intervention with the control group for all the continuous anthropometric and body composition measurements at 60−72 months of age. To compare nutritional status (i.e. using anthropometry as a proxy) grouped as binary (using the respective z-scores < −2) between the intervention and control group, multilevel mixed effect logistic regression models with cluster as a random effect component were used.

In addition, we compared the mean change (gain/loss) of the anthropometric measurements in each of the randomization group from baseline (6−8 months) to 60−72 months age accounting for clustering and also adjusted for the regression-to-the mean. We calculated the regression-to-the mean as the difference between baseline individual anthropometric measurements values and their group baseline (6−8 months) mean [15]. Statistical significance was set at P < 0.05. Statistical analysis was performed using Stata version 15.1 (StataCorp, College Station, TX, USA).

## 3 Results

### 3.1 Study participants

**Fig. 1:**
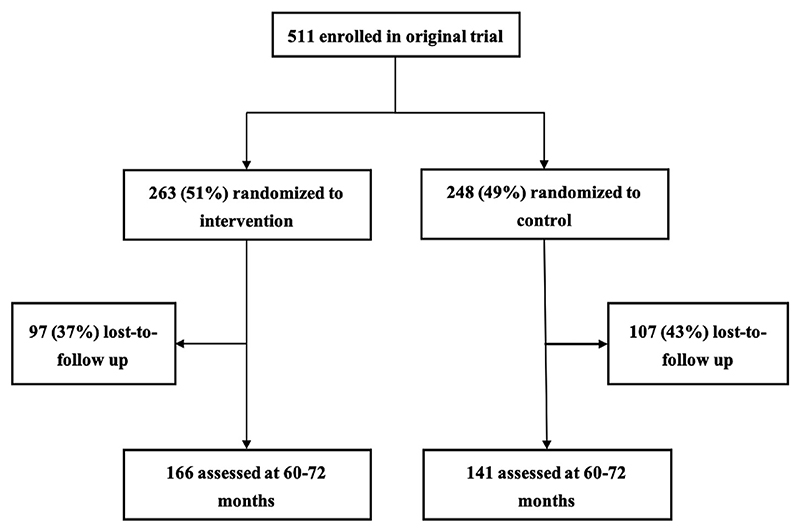
Flow chart showing the enrollment of study participants into the original randomized trial and those attending the current follow-up study.

For this follow-up of the children aged 60−72 months, 166 (mean (SD) age 71.4 (1.9) months) and 141 (mean (SD) age 70.9 (1.7) months) from the intervention and control group (P = 0.47) of the total RCT cohort (n = 511) could be included, respectively (Fig. 1). Among these 511 children, three had died in the intervention and three in the control group (of causes unrelated to the trial). Furthermore, 94 and 104 children in the intervention and control group, respectively, had missing values (did not attend visits or had relocated). Table 1 shows that at the time of randomization to the original RCT, i.e. at baseline when the children were 6−8 months, there were no significant differences in any of the study characteristics, neither among the two study groups (intervention and control) of the RCT nor among the corresponding two study groups in the current follow-up study. We did not detect any intervention-related adverse effects in any of the two study groups.

### 3.2 Anthropometrical data

Since we lacked data from about 40% of the 511 children enrolled into the RCT, we first examined the differences in baseline anthropometrical values between the control and intervention group of the follow-up cohort (Table 2). Importantly, we could not detect any significant difference in baseline HAZ, WAZ, WHZ or MUACZ, strongly indicating that the two groups in the current follow-up study were well balanced at start of the RCT.

Next, we found no significant differences in mean HAZ, WAZ or MUACZ at child age 60−72 months between the control and intervention group (Table 2). In contrast, at child age 60−72 months, those in the intervention group had lower (P = 0.006) WHZ than the controls.

**Fig. 2:**
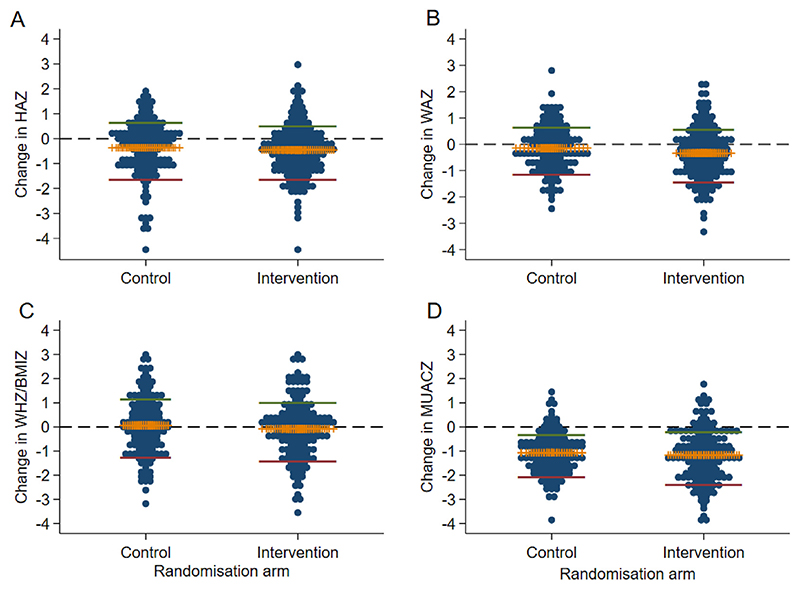
Changes in growth z-scores from baseline (6−8 months) to 60−72 months. (A) Height-for-age z-score (HAZ), (B) Weight-for-age z-score (WAZ), (C) Weight-for-height z-score (WHZ), (D) Mid-upper arm circumference-for-age z-score (MUACZ). The horizontal middle (yellow) bar represents mean change; green and red lines are upper and lower 95% confidence interval of the mean change, respectively. The line Y = 0 (black dashed line) indicates no change between the two time points. Each dot represents the value from one child.

We then examined the mean changes in anthropometrical z-scores from baseline to 60−72 months stratified by study group affiliation. The changes in the four anthropometrical z-scores (HAZ, WAZ, WHZ and MUACZ) were all approximately at or below zero, indicating no average catch-up growth over these 54 months (Fig. 2, Supplementary Table 1). Of notice, the mean change in HAZ from baseline to 60−72 months was negative in both the intervention and the control group. Furthermore, mean HAZ at 60−72 months were < −1.5 in both study groups, i.e. equivalent to linear growth below the 10th percentile on the height-for-age growth chart. Moreover, there were no differences between the mean z-score changes from 6−8 months to 60−72 months between the control and intervention group, even after controlling for regression-to-the mean (all P-values >0.05; Supplementary Table 1).

### 3.3 Height growth velocity

The absolute height growth velocity was not significantly different between the control and intervention group at any of the three sampling time points during the follow-up period (Table 3). As expected, the height growth velocity from baseline was significantly higher in early life (i.e. at child age 12−16 and 20−24 months) compared with data obtained at 36 and at 60−72 months (Supplementary Fig. 1). When stratified by gender, we did not detect any significant change in height growth velocity from baseline over time in any of the two study groups (Supplementary Table 2). Supplementary Fig. 2 depicts the height growth trajectories expressed as HAZ (Supplementary Fig. 2A) and as absolute height values (Supplementary Fig. 2B) during the 54 months’ observation period. Whereas both indices increased more after 36 months compared with the earlier time points, there were no significant changes between the intervention and control groups.

### 3.4 Stunting, wasting and underweight at child age 60−72 months

We then evaluated the anthropometrical z-scores (HAZ, WAZ, WHZ and MUACZ) as markers of nutritional status. At child age 60−72 months, approximately one-third; i.e. 49 (30%) and 43 (31%) (P = 0.86) among children randomized to the intervention and control group, respectively, were stunted. At 60−72 months, wasting was rare; only 1 (0.7%) child from the control group was wasted and none in the intervention group. In contrast, at 60−72 months, more children in the intervention compared with the control group were classified as underweight: 28 (17%) vs. 9 (6.4%); yielding a cluster-adjusted odds ratio of 2.96 (95%CI 1.21 to 7.25; P = 0.02).

### 3.5 Body composition at child age of 60−72 months

To complement our anthropometrical data, we also included measurements of body composition using bioimpedance. Table 4 shows that there were no significant differences in any of the body compartments (fat mass, fat-free mass and body water) between the intervention and control group. In line with this, when stratified per gender we did not detect any significant change in fat mass, fat-free mass or body water from baseline to 60−72 months of age in any of the two study groups (Supplementary Table 3).

## 4 Discussion

Here we present data of a long-term (~6 years) follow-up of a randomized maternal education trial primarily designed to prevent impaired linear growth among small children in rural Uganda. Similar to our previous follow-up studies of this trial cohort, we could not detect any significant effect of the intervention on child anthropometrical z-scores (i.e. HAZ, WAZ, WHZ and MUACZ) [11,12]. In the current follow-up study, we also included measurements of growth velocity, but this parameter was apparently not affected by the education intervention either. Finally, body composition analyses at child age 60−72 months did not reveal any significant differences in body fat mass, body fat-free mass or total body water between the two study groups (intervention and control).

Numerous studies have addressed one or several drivers of impaired growth, but usually with modest or no improvement [16]. Although some community-based interventions have showed significant benefits on child stunting [17,18], we failed to do so with our maternal education intervention, possibly due in part to prenatal influences or insults during the first 6−8 months of life [6,11,12]. Another factor linked to impaired growth has been altered gut bacterial composition in infancy [19], however, our intervention did not impact on the overall gut microbiota [12,20].

Whereas there has been some progress in preventing or reducing impaired linear growth (low HAZ) in many low- and middle-income countries, stunting is still rampant in sub-Saharan Africa [2,3,5,21,22]. The causes of low HAZ and stunting are complex and include poverty, poor sociodemographic characteristics, prevalent maternal depression, inadequate sanitary conditions combined with high infectious burden and lack of micronutrients [23–25]. Increasing evidence indicates that improvement in child stunting prevalence and growth in rural sub-Saharan Africa requires better understanding of the complex underlying mechanisms, since improved access to both nutrition and health care remains inadequate [22,26]. Data also point in the direction that an adverse in utero environment or even transgenerational effects, including epigenetics, are contributing to an increased risk of impaired linear growth [3].

Accumulating evidence suggests that undernutrition and over-weight/obesity share characteristics in terms of trends and underlying determinants [27,28]. In line with this, we found that the maternal education intervention significantly reduced the prevalence of concurrent stunting and overweight among the children when they were 36 months old and later when they were aged 60−72 months [29]. It is intriguing that our maternal education intervention can possibly prevent this combined adverse anthropometrical deficiency several years later after the interventionperiod. However, more research is needed to identify the inherent mechanisms governing such multiple growth impairments, in particular in sub-Saharan Africa where growth trajectories show a worrying tendency towards increasing weight relative to height when children grow older [30]. Notably, we found low prevalence (<5%) of combined stunting and wasting when measured at the same time points as in the current study [29], which is in line with previous global data [22].

We detected a significantly higher fraction of children that were underweight in the intervention group compared with the controls at 60−72 months, but there were no significant changes in the other anthropometric measures or in body composition, and the 95% confidence interval for the estimated odds ratio was also quite wide.

Interestingly, the concept of stunting as a marker for undernutrition has recently been questioned by Scheffler et al. [8,31]. They challenged stature as the tool of choice for detecting undernutrition, and rather emphazised catch-up growth as a better marker. However, there is lack of a clear definition of catch-up growth, ranging from a change in HAZ >0.67, achieving a HAZ above −2 or −1.6, reaching height above the third percentile for height (for age) or even more complex definitions [32–34]. Notwithstanding these methodological challenges, Desmond and Casale, using data from a South African cohort of urban children aged 2−5 years, reported that stunted children exhibited catch-up growth regardless of the definition used, but the prevalence of catch-up varied greatly, from 19% to 93% depending of the definition [35]. Height growth velocity is another measure of linear growth that might detect deviating growth patterns at an earlier time point than impaired growth z-scores, as the latter are only evident after the growth restriction has occurred. This neccesitates the use of reference curves for normal height growth, e.g. those recommended by the WHO [36]. We previously reported that at 36 months, children in the intervention group experienced less growth faltering compared with the controls, even after adjusting for stunting and HAZ at baseline, suggesting that the intervention may have had a protective effect against growth faltering over time [12]. However, no similar changes over time for other anthropometric z-scores were found. Our current data on change in HAZ between baseline and 60−72 months indicate that the children continue to falter in growth as compared to the WHO reference curves. In addition we have now used the height-data from our RCT starting when the children were 6−8 months and until they were aged 60−72 months, to assess height growth velocity (cm/year), as well as change in HAZ. We here report that the height growth velocity up to a child age of 60−72 months was unaffected by the maternal education intervention. As expected, height increased at a faster rate at younger age [up to 20−24 months] compared with older age (up to 60−72 months).

Much focus is on preventing rapid gain of fat mass in infancy to decrease the risk of non-communicable disorders such as cardiovascular disease, diabetes type 2 and the metabolic syndrome, as recently reviewed [37,38]. We therefore included assessment of body composition in the current follow-up study. Similar to our anthropometric findings we could not detect any significant impact of the maternal education intervention on either fat mass, fat-free mass, muscle mass or total body water. The percentage of body fat was nearly 20%, which is within the 50−75 centiles of the body fat reference curves for Caucasian children proposed by McCarthy et al. [39]. Notably, there is currently no bioimpedance-validated reference standards for body composition specifically among African children.

The major strengths of this study are the robust and pragmatic design of the original RCT and the long-follow-up period. The adherence to the intervention was probably adequate as mothers in the intervention group demonstrated more relevant knowledge after the intervention period, compared with the controls [11]. Additionally, despite that the mothers in the intervention group received knowledge of nutrition, food preparation and hygiene, we did not evaluate adherence to the intervention. Limitations include an attrition rate of about 40%, though the current follow-up study was well balanced between the original two study groups; lack of data for dietary intakes, growth, relevant biomarkers and body composition from earlier time-points; and lack of labor data such as gestational length and birth weight. We also lack data on parental anthropometry and child body size at birth, which may impact on postnatal growth. We collected body composition data using bioimpedance, a non-invasive method and more feasible in our challenging remote study setting. Although the bioimpedance method has some limitations, it has proved reliable for body composition analyses in various populations including children from low-resource settings and those with weight loss [40,41]. We have previously shown that our maternal education interveniton led to marked improvements in child developmental outcomes up till 36 months of age [11,12]. However, due to the corona-pandemic we were not able to collect data on developmental outcomes in the current follow-up study.

In conclusion, in this six-year follow-up study of children participating in a randomized maternal education trial, we found no significant impact of the intervention on anthropometrical z-scores, height growth velocity or body composition. This trial cohort should be re-examined when entering into adulthood (i.e. at about 18 years of age), and preferably be supplemented with assessments of various growth biomarkers.

### Statement of authorship

Conceptualization, P.A, G.M, A.C.W. and P.O.I.; Data curation, P.A., X.C. and G.M.; Formal analysis, G.M. and M.N.; Methodology, P.A, M.N., G.M, A.C.W. and P.O.I.; Project administration, P.A., P.O.I. and G.M.; Resources, P.O.I.; Writing-original draft, M.N., A.C.W. and P.O.I.; Writing-review & editing, P.A., M.N., X.C., A.C.W., P.O.I. and G.M.

## Supplementary Material

CONSORT

Figure S1

Figure S2

Supplementary methods

Table S1

Table S2

Table S3

## Figures and Tables

**Table 1:** Study population characteristics at baseline of the original randomized controlled trial.

	Original study (N =511)		Current study (N =307)	
	Control (n =248)	Intervention (n =263)	Control (n =141)	Intervention (n =166)
*Children characteristics*				
Gender-male	123(50)	139(53)	67 (48)	81 (49)
Mean (SD) age (months)	7.3 ± 0.9	7.4 ± 0.8	7.2 ± 0.9	7.4 ± 0.9
Underweight	36 (15)	25 (9.5)	17(12)	16 (9.6)
Wasting	12 (4.8)	12 (4.6)	5 (3.6)	8 (4.8)
Stunting	70 (28)	55(21)	40 (28)	34 (20)
Exclusive breastfeeding for 6 months				
Yes	178 (72)	184(70)	100(71)	107 (64)
No	70 (28)	79 (30)	41 (29)	59 (36)
Breastfeeding frequency				
≥8 times/day	171 (69)	170 (65)	99 (70)	109 (67)
<8 times/day	77(31)	93 (35)	42 (30)	57 (34)
Illness at recruitment				
Yes	71 (29)	94 (36)	48 (34)	45 (27)
No	177(71)	169 (64)	93 (66)	121 (73)
*Maternal characteristics*				
Median (IQR) maternal age (years)^a^	26 (22−30)	25 (21−30)	27 (22−30)	25 (21−30)
Level of education				
None/primary	166(70)	173 (66)	95 (67)	110(66)
Lower secondary	62 (25)	64 (24)	38 (27)	45 (27)
Tertiary	20 (8.1)	26 (9.9)	8 (5.7)	11 (6.6)
Number of biological children				
<5	184 (74)	187 (71)	91 (65)	116(70)
≥5	64 (26)	76 (29)	50 (35)	50 (30)
*Household-level characteristics*				
Median (IQR) household head age (years)	30 (25−38)	30 (25−36)	30 (25−39)	30 (25−35)
Level of education				
None/primary	138(56)	138(52)	77 (55)	82 (49)
Lower secondary	80 (32)	78 (30)	47 (33)	55 (33)
Tertiary	30(12)	47 (18)	17(12)	29(18)
Household size				
3 to 5	139(56)	150(57)	72 (51)	96 (58)
6 to 10	109(44)	113(43)	69 (49)	70 (42)
Median (IQR) poverty score	49 (39−57)	49 (40−57)	49 (40−57)	49 (40−55)

Values are n (%) unless otherwise stated.

a 7 missing records (4 in intervention and 3 in control group). There were no differences (P > 0.05) in any of the characteristics between the two study groups, neither for the original randomized controlled trial cohort nor for the follow-up cohort. Underweight was defined as weight-for-age z score < −2 standard deviations (SD) below the median of the WHO child growth standard; wasting as weight-for-height z-score < −2 SD for children 6−8 months and as BMI-for-age z-score < −2 SD for children aged 60−72 months; and stunting as height-for-age z-score < −2 SD. IQR, interquartile range.

**Table 2:** Child anthropometrical z-scores at baseline and at 60−72 months of age.

	Control (n =141)	Intervention (n =166)	Mean difference (95% CI)^a^	P-value^b^
*HAZ*				
Baseline (6−8 months)	−1.19 (−1.47 to −0.91)	−1.08 (−1.34 to −0.82)	−0.11 (−0.43 to 0.21)	0.45
60−72 months	−1.56 (−1.90 to −1.22)	−1.54 (−1.87 to−1.21)	−0.02 (−0.41 to 0.37)	0.87
*WAZ*				
Baseline (6−8 months)	−0.68 (−0.93 to −0.42)	−0.68 (−0.92 to −0.45)	0.006 (−0.28 to 0.29)	0.96
60−72 months	−0.83 (−1.03 to −0.63)	−1.02 (−1.21 to −0.84)	0.20 (−0.03 to 0.42)	0.08
*WHZ*				
Baseline (6−8 months)	0.22 (−0.13 to 0.56)	0.06 (−0.27 to 0.38)	0.16 (−0.23 to 0.55)	0.38
60−72 months	0.27 (0.11−0.44)	−0.02 (−0.18 to 0.13)	0.30(0.11−0.48)	0.006
*MUACZ*				
Baseline (6−8 months)	0.37 (0.01−0.73)	0.22 (−0.13 to 0.57)	0.15 (−0.27 to 0.57)	0.44
60−72 months	−0.77 (−0.99 to −0.55)	−0.95 (−1.16 to −0.73)	0.18 (−0.08 to 0.43)	0.07

Values are means (95% confidence intervals). CI, confidence interval; HAZ, height-for-age z-score; WAZ, weight-for-age z-score; WHZ, weight-for-height z-score. MUACZ, mid-upper arm circumference z-score.

a Mean difference is the cluster-adjusted difference in means between the control and intervention and group.

b P-values from multilevel regression models with cluster as random intercept.

**Table 3:** Child height growth velocity from baseline to 60−72 months.

Time-point	Control (n = 128)	Intervention (n = 166)	Mean difference (95% CI)^a^	P-value^b^
12−16 months	12.66 (10.58−14.74)	12.41 (10.38−14.44)	0.25 (−2.16−2.66)	0.81
20−24 months	10.70 (9.17−12.22)	11.27 (9.78−12.77)	−0.58 (−2.35−1.20)	0.40
36 months	8.95 (8.27−9.62)	9.41 (8.74−10.08)	−0.46 (−1.25−0.33)	0.14
60−72 months	7.78 (7.37−8.19)	7.84 (7.44−8.24)	−0.06 (−0.54−0.42)	0.71

We calculated the difference between height at each time-point (12−16, 20−24, 36 and 60−72 months) and baseline height values divided by the follow-up time. The obtained values are given as cm/year and presented as mean (95% confidence interval).

a Mean difference is the cluster-adjusted difference in means between the control and intervention and group.

b P-values from multilevel regression models with the cluster as random intercept. CI, confidence interval.

**Table 4:** Body composition among the children aged at 60−72 months.

Body composition	Control (n = 128)	Intervention (n = 166)	Mean difference (95% CI)^a^	P-value^b^
Body fat mass (kg)	3.6 (3.4−3.8)	3.4 (3.2−3.6)	0.17 (−0.08 to 0.41)	0.07
Body fat (%)	19.5 (18.4−20.6)	19.2 (18.1−20.2)	0.37 (−0.89 to 1.64)	0.37
Body fat-free mass (kg)	14.6(14.2−14.9)	14.4 (14.0−14.7)	0.16 (−0.27 to 0.60)	0.38
Body muscle mass (kg)	13.7 (13.4−14.1)	13.5 (13.2−13.9)	0.20 (−0.21 to 0.62)	0.25
Total body water (l)	10.7 (10.4−11.0)	10.5 (10.3−10.8)	0.17 (−0.15 to 0.48)	0.22
Total body water (%)	58.8 (58.1−59.6)	59.1 (58.4−59.8)	−0.27 (−1.16 to 0.62)	0.37

Values are given as mean (95% confidence interval) unless otherwise specified.

a Mean difference is the cluster-adjusted difference in means between the control and intervention and group.

b P-values from multilevel regression models with the cluster as random intercept. CI, confidence interval.
