# Supplementary material for: Randomized Control Trials Longitudinal assessments of child growth: A six-year follow-up of a cluster-randomized maternal education trial
Source: Clin Nutr. Author manuscript; Available in PMC 2022 Sep 7. (PMC7613314; doi:10.1016/j.clnu.2021.08.007)

**Supplementary Figure 1.** Height increments from baseline (6–8 months of age) in cm per year at each of the follow-up time points. We found a significant linear decline trend in height velocity from 12–16 months to 60–72 months among children randomized to intervention (linear regression coefficient -1.56 and linear test *P*-value = 0.004) and control (linear regression coefficient -1.64 and linear test *P*-value = 0.006). Values are mean (95% confidence interval).


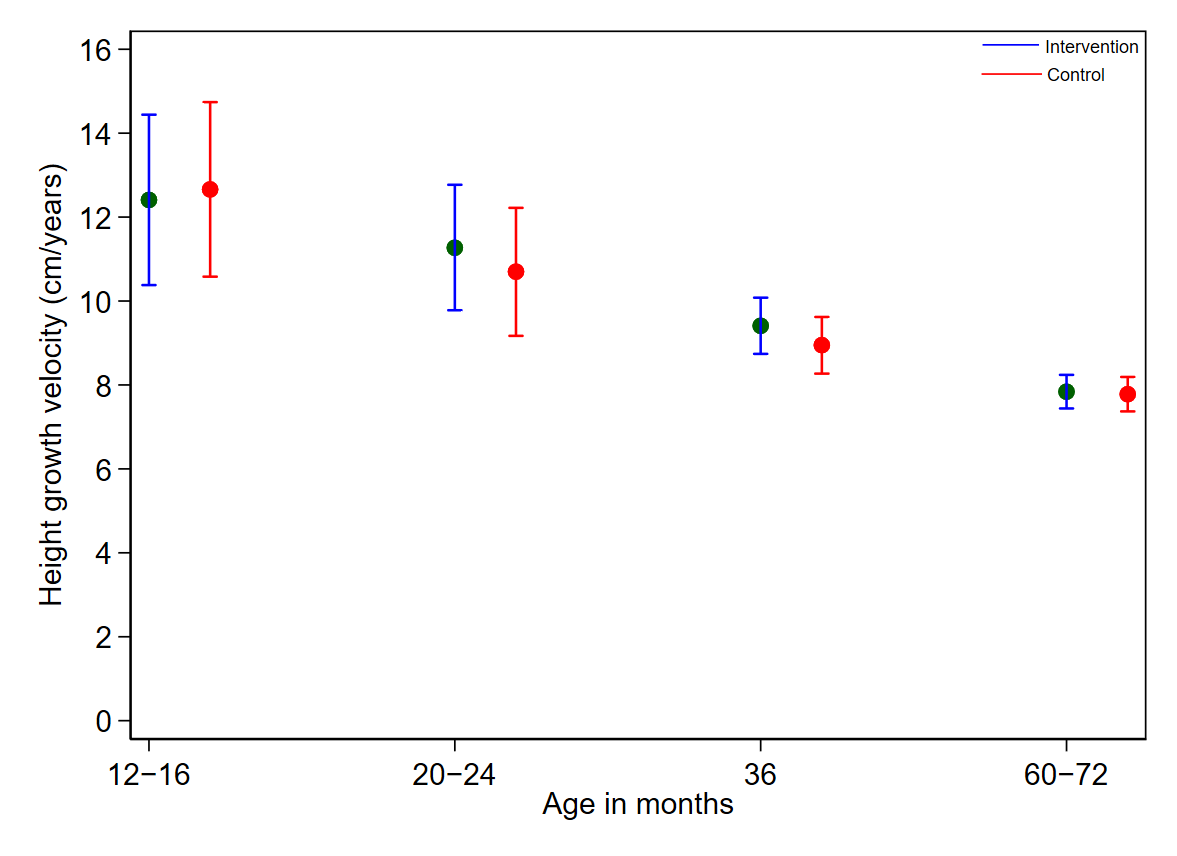

Supplement: Figure S1 [file EMS152533-supplement-Figure_S1.docx]
