# Supplementary material for: Randomized Control Trials Longitudinal assessments of child growth: A six-year follow-up of a cluster-randomized maternal education trial
Source: Clin Nutr. Author manuscript; Available in PMC 2022 Sep 7. (PMC7613314; doi:10.1016/j.clnu.2021.08.007)

**Supplemental Figure 2.** Height-for-age z-score (A) and height (B) trajectory from baseline (6–8 months of age) to 60–72 months. Values are mean (95% confidence interval).

**A**


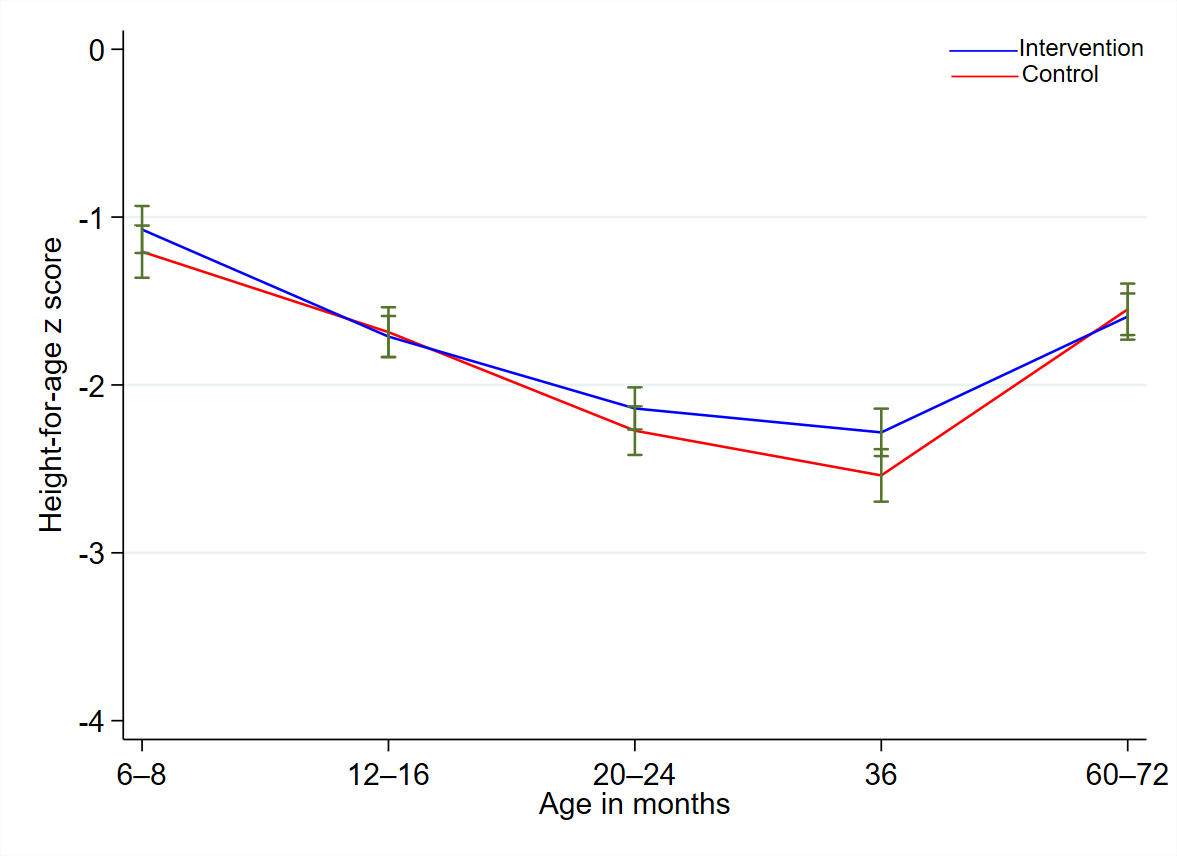


**B**


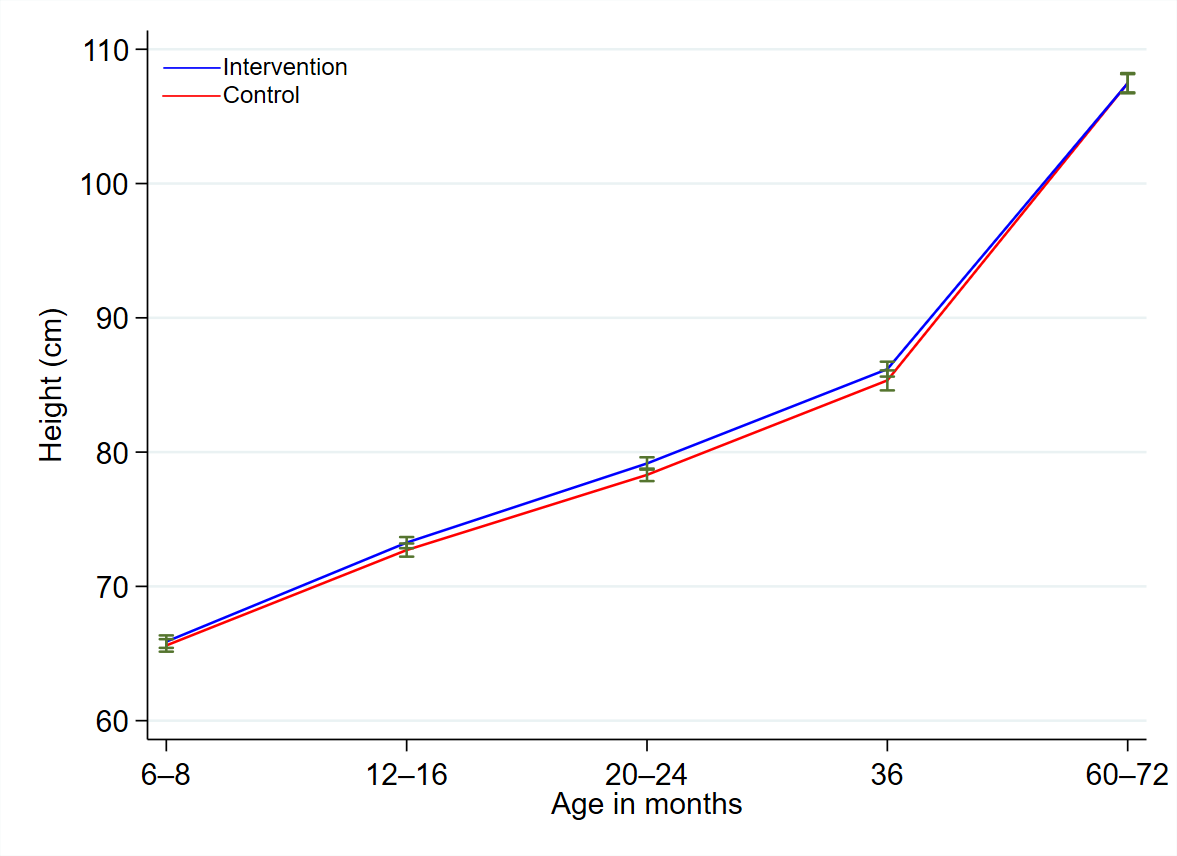

Supplement: Figure S2 [file EMS152533-supplement-Figure_S2.docx]
