# Supplementary material for: Randomized Control Trials Longitudinal assessments of child growth: A six-year follow-up of a cluster-randomized maternal education trial
Source: Clin Nutr. Author manuscript; Available in PMC 2022 Sep 7. (PMC7613314; doi:10.1016/j.clnu.2021.08.007)
Supplement: Table S1 [file EMS152533-supplement-Table_S1.docx]

**Supplementary Table 1.** Changes in child anthropometry from baseline (6-8 months) to 60-72 months in each study group

| Anthropometrical  z-score | Change from baseline (6−8 months) to 60−72 months^1^ | | Mean difference in change (95% CI)^2^ | *P*-value* |
| --- | --- | --- | --- | --- |
|  | Control  (n=141) | Intervention  (n=166) |  |  |
| HAZ | -0.37 (-0.63 to -0.10) | -0.46 (-0.70 to -0.21) | 0.09 (-0.21 to 0.39) | 0.88 |
| WAZ | -0.15 (-0.37 to 0.07) | -0.34 (-0.54 to -0.14) | 0.19 (-0.06 to 0.44) | 0.06 |
| WHZ | 0.06 (-0.34 to 0.45) | -0.08 (-0.46 to 0.30) | 0.14 (-0.31 to 0.59) | 0.38 |
| MUACZ | -1.14 (-1.53 to -0.74) | -1.17 (-1.55 to -0.78) | 0.03 (-0.43 to 0.49) | 0.14 |
| ^1^The values are the cluster-adjusted means (95% confidence intervals) for change from baseline to 60-72 months for each of the two study groups (control and intervention).  ^2^The values are the cluster-adjusted difference in means (95% confidence intervals) between the control and intervention group, **P*-values are derived from multilevel regression models controlling for regression-to-the mean with cluster as random concept. HAZ, height-for-age z-score; WAZ weight-for-age z-score; WHZ, weight-for-height z-score; MUACZ, mid-upper arm circumference-for-age z-score. | | | | |
