# Supplementary material for: Randomized Control Trials Longitudinal assessments of child growth: A six-year follow-up of a cluster-randomized maternal education trial
Source: Clin Nutr. Author manuscript; Available in PMC 2022 Sep 7. (PMC7613314; doi:10.1016/j.clnu.2021.08.007)
Supplement: Table S2 [file EMS152533-supplement-Table_S2.docx]

**Supplementary Table 2.** Height increments from baseline (6–8 months of age) to 60-72 months in each study group stratified by gender

| Time | Height growth velocity (cm/years) | | | | | |
| --- | --- | --- | --- | --- | --- | --- |
|  | Control (n = 174) | | | Intervention (n = 198) | | |
|  | Male (n = 86) | Female (n = 88) | *P*-value | Male (n = 103) | Female (n = 95 ) | *P*-value |
| 12–16 months | 12.9 (10.1–15.7) | 12.4 (9.6–15.2) | 0.76 | 12.7 (9.6–15.8) | 12.1 (8.8–15.3) | 0.75 |
| 20–24 months | 10.5 (7.9–13.1) | 10.9 (8.3–13.5) | 0.77 | 11.6 (9.4–13.9) | 10.9 (8.4–13.3) | 0.56 |
| 36 months | 9.2 (8.3–10.1) | 8.6 (7.7–9.5) | 0.39 | 9.7 (7.5–11.8) | 9.3 (7.0–11.6) | 0.74 |
| 60–72 months | 7.5 (6.9–8.2) | 8.0 (7.4–8.6) | 0.34 | 7.9 (7.3–8.5) | 7.8 (7.2–8.4) | 0.87 |
| Values are given as mean (95% confidence interval). *All *P*-values comparing male and females in each arm were >0.05. The *P*-values were from multilevel regression models with the cluster as random intercept. | | | | | | |
