# Supplementary material for: Randomized Control Trials Longitudinal assessments of child growth: A six-year follow-up of a cluster-randomized maternal education trial
Source: Clin Nutr. Author manuscript; Available in PMC 2022 Sep 7. (PMC7613314; doi:10.1016/j.clnu.2021.08.007)
Supplement: Table S3 [file EMS152533-supplement-Table_S3.docx]

**Supplementary Table 3.** Body composition among the children aged 60-72 months stratified by gender

| Body composition | Control (n = 128) | | | | | | Intervention (n = 166) | | | | | |  |
| --- | --- | --- | --- | --- | --- | --- | --- | --- | --- | --- | --- | --- | --- |
|  | Male (n = 61) | | Female (n = 67) | | *P*-value | | Male (n = 81) | | Female *(*n *=* 85) | | *P-*value | |  |
| Body fat mass (kg) | | 3.64 (3.15–4.13) | | 3.56 (3.10–4.03) | | 0.38 | | 3.39 (3.11–3.66) | | 3.48 (3.22–3.75) | | 0.40 | |
| Body fat (%) | | 19.7 (17.7–21.7) | | 19.4 (17.5–21.2) | | 0.34 | | 18.6 (16.8–20.5) | | 19.6 (17.9–21.4) | | 0.23 | |
| Body fat-free mass (kg) | | 14.6 (14.1–15.2) | | 14.5 (14.0–15.0) | | 0.66 | | 14.7 (14.2–15.2) | | 14.1 (13.6–14.8) | | 0.11 | |
| Body muscle mass (kg) | | 13.8 (13.2–14.3) | | 13.7 (13.2–14.2) | | 0.84 | | 13.8 (12.9–14.7) | | 13.3 (12.4–14.2) | | 0.33 | |
| Total body water (l) | | 10.7 (10.3–11.2) | | 10.6 (10.3–11.0) | | 0.58 | | 10.8 (10.2–11.4) | | 10.3 (9.70–10.9) | | 0.32 | |
| Total body water (%) | | 58.7 (56.8–60.6) | | 59.0 (57.2–60.8) | | 0.30 | | 59.4 (57.9–60.9) | | 58.8 (57.3–60.3) | | 0.27 | |
| Values are given as mean (95% confidence interval). The *P*-values were from multilevel regression models with the cluster as random intercept. | | | | | | | | | | | | |  |
